# Effects of Mindfulness-Based Stress Reduction on Depression in Adolescents and Young Adults: A Systematic Review and Meta-Analysis

**DOI:** 10.3389/fpsyg.2018.01034

**Published:** 2018-06-21

**Authors:** Xinli Chi, Ai Bo, Tingting Liu, Peichao Zhang, Iris Chi

**Affiliations:** ^1^College of Psychology and Sociology, Shenzhen University, Shenzhen, China; ^2^Shenzhen Key Laboratory of Affective and Social Cognitive Science, Shenzhen University, Shenzhen, China; ^3^Silver School of Social Work, New York University, New York, NY, United States; ^4^Department of Sociology, Wuhan University, Wuhan, China; ^5^Research Center of Modern Psychology, Department of Philosophy, Wuhan University, Wuhan, China; ^6^Suzanne Dworak-Peck School of Social Work, University of Southern California, Los Angeles, CA, United States

**Keywords:** mindfulness-based stress reduction, adolescents, young adults, randomized controlled trials, depressive symptoms, meta-regression

## Abstract

**Background:** Mindfulness as a positive mental health intervention approach has been increasingly applied to address depression in young people. This systematic review and meta-analysis evaluated the effects of mindfulness-based stress reduction (MBSR) in the treatment of depression among adolescents and young adults.

**Methods:** Electronic databases and references in articles were searched. Randomized controlled trials (RCTs) evaluating MBSR and reporting outcomes for depressive symptoms among young people aged 12 to 25 years were included. Data extraction and risk of bias assessment were conducted by two reviewers independently. Hedges’ *g* with a 95% confidence interval was calculated to represent intervention effect.

**Results:** Eighteen RCTs featuring 2,042 participants were included in the meta-analysis. Relative to the control groups (e.g., no treatment, treatment as usual, or active control), MBSR had moderate effects in reducing depressive symptoms at the end of intervention (Hedges’ *g* = −0.45). No statistically significant effects were found in follow-up (Hedges’ *g* = −0.24) due to a lack of statistical power. Meta-regression found that the average treatment effect might be moderated by control condition, treatment duration, and participants’ baseline depression.

**Conclusion:** MBSR had moderate effects in reducing depression in young people at posttest. Future research is needed to assess the follow-up effects of MBSR on depressive symptoms among adolescents and young adults.

## Introduction

Depression among adolescents and young adults is a serious public health problem. Depression affects approximately 8 to 20% of adolescents before the age of 18 worldwide (Naicker et al., 2013). The mean prevalence of depressive symptoms among college students is 30.6% globally (Ibrahim et al., 2013). Recent studies found that major depressive episodes have increased significantly among U.S. adolescents aged 12 to 17, from a 12-month prevalence of 8.7% in 2005 to 12.7% in 2015 (Mojtabai et al., 2016; National Institution of Mental Health, 2017). In China, the prevalence of depressive symptoms among university students aged 16 to 35 years was estimated to be 11.7% (Chen et al., 2013). Depression is a serious threat to the physical and psychological health of adolescents and young adults and may result in negative social and behavioral consequences, including academic failure (Quiroga et al., 2013), social disorders (Verboom et al., 2014), drug use (Curry et al., 2012), and suicide (Tuisku et al., 2014). Furthermore, depression in adolescence may have a continuous effect that causes physical and mental disorders and behavioral problems in adulthood (Dunn and Goodyer, 2006).

Given this disturbing situation, the problem of youth depression has attracted attention from practitioners and scholars. Many intervention methods have been implemented to prevent and reduce depression in young people. While antidepressant medication is reserved for treating severe depression, psychotherapy has been widely used to treat mild to moderate depression (Cheung et al., 2007; McDermott et al., 2010). Established psychotherapies such as cognitive behavioral therapy and interpersonal psychotherapy were found to be the most effective for treating adolescent depression (Zhou et al., 2015). Emerging treatments such as mindfulness-based interventions have also gained popularity and a supportive evidence base regarding treating youth depression in the last decade (Kallapiran et al., 2015; Zoogman et al., 2015; Felver et al., 2016).

Mindfulness is derived from Eastern meditation practices and Buddhist philosophy. It refers to “bringing one’s complete attention to the present experience on a moment-to-moment basis” (Marlatt and Kristeller, 1999, p. 68). Mindfulness-based interventions aim to help individuals be aware of their present emotion, pay attention to the present task at hand, and promote inner peace and happiness. Mindfulness-based interventions were initially designed for adults and have been extended and adapted to children, adolescents, and young adults for a variety of clinical problems and to promote youth well-being. Well-established standardized mindfulness-based interventions include mindfulness-based stress reduction (MBSR), mindfulness-based cognitive therapy (MBCT), acceptance and commitment therapy (ACT), and dialectical behavior therapy (DBT; Hunot et al., 2010; Chiesa and Malinowski, 2011). Among these interventions, MBSR was the earliest to be developed and is the most frequently used intervention method.

MBSR was originally developed to help patients with physical illness to deal with pain, stress, and negative emotions in behavioral medicine settings (Kabat-Zinn, 1982, 1990). Nowadays, MBSR is widely used in the general population for stress, anxiety, and depression mitigation (Virgili, 2015). By cultivating self-awareness and an attitude of openness and acceptance, MBSR may help individuals calm their mind and body, make better judgments in life, and enhance self-capability to cope with various stressful situations (Carmody et al., 2009). The standard MBSR program features an 8-week course with a 2- to 2.5-h weekly session; a 1-day retreat (6-h mindfulness practice) between Sessions 6 and 7; and 45-min of daily homework. MBSR provides formal mindfulness practice training, including mindfulness meditation, body scans, and yoga movement (Zainal et al., 2013). Additionally, MBSR has been adapted for adolescents to practice mindfulness. For example, Learning to BREATHE (L2B) with six sessions (50-min course, 1 week) is a universal school-based prevention program designed specifically for adolescents to deal with multiple emotional problems, including depression, and strengthen emotional regulation (Broderick, 2013).

Many studies found promising results of mindfulness in reducing individuals’ psychological distress and improving emotional reactivity, behavioral regulation, subjective well-being, and quality of life among young adults (Keng et al., 2011). Additionally, mindfulness-based interventions for adolescents and young adults often take place in school settings, serving as a tool for positive education (Shapiro et al., 2008; Meiklejohn et al., 2012). Existing intervention studies showed that mindfulness-based intervention was associated with students’ increased positive emotion and decreased negative affect (Huppert and Johnson, 2010; Nidich et al., 2011; Maynard et al., 2017). In addition to general school settings, mindfulness-based interventions were also applied in clinical settings to promote mental health of young people with psychopathological conditions such as anxiety and depressive disorders (Cotton et al., 2014; Malboeuf-Hurtubise et al., 2017).

Several systematic reviews have demonstrated some evidence that mindfulness-based interventions including MBSR can reduce psychological symptoms, including depressive symptoms among adolescents (Kallapiran et al., 2015; Zoogman et al., 2015; Felver et al., 2016). Despite the promising results yielded by these studies, varied types of mindfulness practices (e.g., MBSR, MBCT, ACT), different outcomes (e.g., depression, anxiety, emotional problems), different study designs [e.g., non-randomized controlled trials (RCTs) and RCTs] were blended by previous reviews, which masked the evidence of the specific treatment effect of MBSR for depressive symptoms among young people. Because of its wide use among young people in recent years, it is necessary to examine the effect of MBSR on depression in this population. Therefore, we intended to explore the effect of MBSR on depression among adolescents and young adults by conducting a systematic review and meta-analysis of relevant RCTs.

## Materials and Methods

This study was conducted in accordance with the PRISMA guidelines, which provides detailed guidance for the preferred reporting style of systematic reviews and meta-analyses (Moher et al., 2009).

### Inclusion Criteria

Studies were included in the systematic review according to the following eligibility criteria.

#### Type of Studies

Randomized controlled trial design was a key eligibility criterion to screen studies examining MBSR interventions for depression among adolescents and young adults. In this systematic review, we included studies written in English and Chinese language.

#### Type of Participants

The systematic review included studies with adolescents and young adults aged 12 to 25 years old. Participants who were clinically diagnosed as depressed using any diagnostic criterion, such as ICD-10 (Office of the Secretary, Health and Human Services [HHS], 2009) or DSM-5 (American Psychiatric Association, 2013) diagnostic criterion or who scored above a cutoff score on a depression rating scale (e.g., Center for Epidemiologic Studies Depression Scale ≥ 16) were eligible for inclusion. Due to the potentially small number of trails available in this field, we also included studies that used nonclinical samples (i.e., youth with depressive symptoms not reaching the level of clinical diagnosis) to detect the efficacy of MBSR in reducing depressive symptoms.

#### Type of Interventions

Experimental groups involved MBSR or adapted MBSR programs conducted according to the manual by Kabat-Zinn (1990). Control groups featured no treatment (e.g., no treatment in a control group or waitlist control group), treatment as usual (TAU; e.g., standard medical treatment or other standard practices), or active control condition with any nontherapeutic activities (e.g., health education or relaxing activities).

#### Outcome Measures

Change in depressive symptoms was the primary outcome, as measured using depression rating scales, such as the Hamilton Rating Scale for Depression (Hamilton, 1960), the Center for Epidemiologic Studies Depression Scale (McDowell and Newell, 1996), the Beck Depression Inventory-II (Beck et al., 1996), the Hospital Anxiety and Depression Scale (Zigmond and Snaith, 1983), the Depression Anxiety Stress Scales (Lovibond and Lovibond, 1995), and the Symptom Checklist-90 (Derogatis et al., 1973).

### Search Methods

Electronic databases including PubMed, PsycINFO (Ovid), CINAHL, Web of Science, Embase, ProQuest, Cochrane Library, China National Knowledge Infrastructure, and Wangfang Data were searched to identify studies from the first available year to April 2018. We used following search terms: (“mindfulness-based stress reduction” OR MBSR) AND depress^∗^ AND (adolescen^∗^ OR youth OR student OR “young people” OR “young adult”). In addition, reference lists of selected articles and related reviews were hand searched.

### Data Collection and Analysis

#### Selection of Studies

Identified records were exported into Endnote X6 to remove duplicates and screen titles and abstracts (Thomson Reuters, 2011). After removing duplicates, two independent reviewers conducted title and abstract screening of the remaining records. Then the two reviewers independently screened full-text articles that passed the title and abstract screening. Finally, studies meeting the inclusion criteria were included for the systematic review and meta-analysis.

#### Data Extraction

A data extraction sheet was developed based on the data collection form for intervention reviews recommended by Cochrane’s guidelines for systematic reviews (Higgins and Green, 2008). The data extraction sheet includes study characteristics (e.g., study source, study design, sample inclusion and exclusion criteria, sample characteristics, intervention component, duration and frequency, setting and interventionist, control condition, outcome measures, and follow-up timing) and effect size data (e.g., sample size, posttest and follow-up means, standard deviations, or standard errors for the means). Data were extracted by two reviewers independently. If the two reviewers disagreed, a third reviewer was consulted to achieve consensus.

#### Assessment of Risk of Bias

Included studies were assessed for risk of bias using the Cochrane Collaboration’s risk of bias tool as described in the *Cochrane Handbook for Systematic Reviews of Interventions* (Higgins and Green, 2008). This assessment was performed independently by two reviewers. In the event of disagreements regarding the assessment of studies, a third reviewer was consulted. The risk of bias assessment covered the following items: allocation concealment, random sequence generation, blinding of participants and personnel, blinding of outcome assessment, incomplete outcome data, and reporting bias. Each item was rated as high, low, or unclear, with an explanation. The results of the meta-analysis were interpreted with consideration of the risk of bias of the studies. We used Review Manager 5.3 (Cochrane Collaboration, 2014) to assess the risk of bias in included studies and present the results graphically.

#### Data Synthesis

Because of the variation in study characteristics (e.g., participant characteristics, geographical region), we assumed that the true effect size may vary from study to study. Thus, posttest effects were synthesized based on random-effects modeling (Borenstein et al., 2010). We used Comprehensive Meta-Analysis software version 2.0 to synthesize the effect size of continuous data using Hedges’ *g* with 95% confidence intervals (CIs) and to generate the forest plot (Borenstein et al., 2005). Hedges’ *g* provides unbiased estimates through small sample size correction (Hedges and Olkin, 1985). *I*^2^ and *T*^2^ statistics were used to examine overall heterogeneity between studies. *I*^2^ indicates the proportion of the variation in observed effects that is due to the variation in true effects (Higgins et al., 2003). For standardized mean differences, the prediction intervals (i.e., an approximate 95% range of underlying effects) can be obtained by creating an interval from two times *T* below and above the random-effects pooled estimate (Higgins and Green, 2008). Potential sources of heterogeneity were explored using sensitivity analyses and meta-regression.

#### Meta-Regression

Meta-regression was performed to explore whether the treatment effect of MBSR varied based on the following: (a) control condition (e.g., no treatment or TAU versus active control), (b) participant age group (i.e., adolescent versus young adult), (c) baseline depression diagnosis (i.e., participants with a formal depression diagnosis at baseline versus participants with depressive symptoms only), and (d) duration of the treatment (i.e., less than 8 weeks versus 8 weeks or more). Robust variance estimation in meta-regression was used to synthesize the follow-up treatment effect size and to conduct moderator analysis in Stata (StataCorp, 2015). Several studies had multiple follow-ups, and thus these data were not statistically independent from one another. Robust variance estimate allows for the inclusion of dependent effect size estimates (Hedges et al., 2010; Tanner-Smith and Tipton, 2014). *Post hoc* power analysis was conducted for meta-analysis of average effect size and meta-regression to determine if nonsignificant results were due to low statistical power (Hedges and Pigott, 2001, 2004). We decided that a moderate effect size of 0.30 was important to detect, and that effect sizes less than 0.30 were too small to matter.

#### Assessment of Publication Bias

A funnel plot was used to explore the presence of publication bias. We included a formal test of funnel plot asymmetry as outlined by Egger et al. (1997) to examine the association between the overall estimated posttest intervention effect and the standard error of the intervention effect. The funnel plot trim-and-fill method described by Duval and Tweedie (2000) was used to adjust for possible bias in the overall effect size by accounting for effect sizes from the estimated number of missing studies.

## Results

### Description of Studies

#### Results of the Search

**FIGURE 1:**
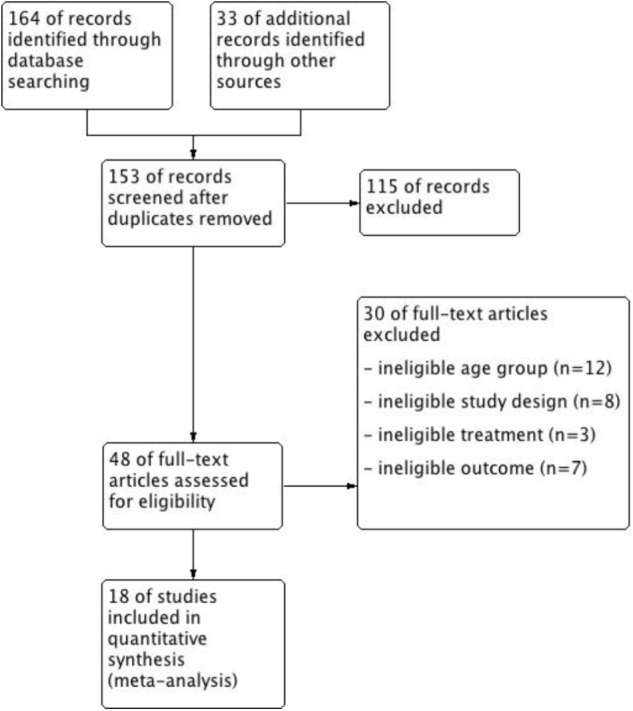
Search flow: Trials identified and search process.

The search process is summarized in **Figure 1** using a PRISMA flow diagram (Moher et al., 2009). Review Manager 5.3 (Cochrane Collaboration, 2014) was used to produce the figure.

#### Included Studies

##### Study origins and year of publication

The characteristics of the 18 included studies examining MBSR for prevention and intervention of depression in young people are summarized in **Table 1**. These studies were conducted in five countries: United States (*n* = 12; Shapiro et al., 1998; Jimenez, 2008; Biegel et al., 2009; Hindman, 2013; Sibinga et al., 2013, 2016; McIndoo, 2015; Bluth et al., 2016; Dvořáková et al., 2017; Freedenberg et al., 2017; Hazlett-Stevens and Oren, 2017; Shomaker et al., 2017), Korea (*n* = 2; Kang et al., 2009; Song and Lindquist, 2015), United Kingdom (*n* = 1; Raes et al., 2014), Australia (*n* = 1; Johnson et al., 2016), Thailand (*n* = 1; Aeamla-Or, 2015), and China (*n* = 1; Huang et al., 2015). The 18 studies were published between 1998 and 2017. Fourteen studies were published journal articles (Shapiro et al., 1998; Biegel et al., 2009; Kang et al., 2009; Sibinga et al., 2013, 2016; Raes et al., 2014; Huang et al., 2015; Song and Lindquist, 2015; Bluth et al., 2016; Johnson et al., 2016; Dvořáková et al., 2017; Freedenberg et al., 2017; Hazlett-Stevens and Oren, 2017; Shomaker et al., 2017) and four studies were dissertations (Jimenez, 2008; Hindman, 2013; Aeamla-Or, 2015; McIndoo, 2015).

**Table 1:** Characteristics of included studies.

Author, year (country)	Participants	Experimental Group			Control Group			Follow-Up Time	Outcome Measure
		*n*	Age, *M* (*SD*), or Range	Intervention	*n*	Age, *M* (*SD*), or Range	Intervention		
Aeamla-Or (2015; Thailand)	Nursing students	63	19.27 (0.79)	MBSR (8 weeks)	64	19.08 (0.93)	None	16 and 32 weeks	CES-D
Biegel et al. (2009; United States)	Adolescent psychiatric outpatients	50	15.7 (1.13)	MBSR (8 weeks)	52	15.0 (1.19)	TAU	12 weeks	SCL-90
Bluth et al. (2016; United States)	Ethnically diverse at-risk adolescents	14	16.8 (1.3)	Learning to BREATHE (11 weeks)	13	17.2 (1.1)	Substance abuse class	None	SMFQ
Dvořáková et al. (2017; United States)	First-year college students	55	18.2 (0.4)	Learning to BREATHE (6 weeks)	54	18.2 (0.4)	None	None	PHQ
Freedenberg et al. (2017; United States)	Adolescents with cardiac diagnoses	30	15.1 (1.8)	MBSR (6 weeks)	22	14.5 (1.6)	Live video conference	None	HADS
Hazlett-Stevens and Oren (2017; United States)	Undergraduate and graduate students	47	22.1 (4.7)	MBSR (10 weeks)	45	22.1 (4.7)	None	None	DASS-21
Hindman (2013; United States)	Undergraduate and graduate students	13	20.92 (n/a)	MBSR (6 weeks)	10	23.80 (n/a)	None	None	DASS-21
Huang et al. (2015; China)	Adolescent inpatients	60	13–18	MBSR (8 weeks)	60	13–18	TAU	None	HAMD
Johnson et al. (2016; Australia)	Young adolescents	165	13.63 (0.43)	Modified MBSR (8 weeks)	128	13.63 (0.43)	None	12 weeks	DASS-21
Jimenez (2008; United States)	University students	61	19.81 (n/a)	MBSR (4 weeks)	59	19.81 (n/a)	Relaxing training	12 and 24 weeks	CES-D
Kang et al. (2009; Korea)	Nursing students	21	22.69 (1.49)	MBSR (8 weeks)	20	22.25 (0.86)	None	None	BDI
McIndoo (2015; United States)	Depressed college students	20	19.3 (1.9)	MBSR(4 weeks)	14	19.0 (1.5)	None	4 weeks	BDI-II &HRSD
Raes et al. (2014; United Kingdom)	Secondary school students	201	15.4 (1.2)^a^	Modified MBSR (8 weeks)	207	15.4 (1.2)	None	24 weeks	DASS-21
Shapiro et al. (1998; United States)	Premedical students and first- and second-year medical students	37	n/a^b^	MBSR (8 weeks)	39	n/a	None	None	SCL-90
Shomaker et al. (2017; United States)	Adolescent girls	17	15.01 (1.68)	Learning to breathe (6 weeks)	16	14.97 (1.75)	Cognitive behavioral program	24 weeks	CES-D
Sibinga et al. (2013; United States)	Middle school students	22	12.5 (n/a)	MBSR (12 weeks)	19	12.5 (n/a)	Health education	None	SCL-90
Sibinga et al. (2016; United States)	Primary and middle school students	159	12.0 (n/a)	MBSR (12 weeks)	141	12.0 (n/a)	Health education	None	CDI-S
Song and Lindquist (2015; Korea)	Nursing students	21	19.6 (1.7)	MBSR (8 weeks)	23	19.5 (2.0)	None	None	DASS-21

*^a^Mean age for whole group of participants without differentiating between experimental and control groups.*

*^b^Participants composed of premedical students and first- and second-year medical students.*

*BDI-II, Beck Depression Inventory; CDI-S, Children’s Depression Inventory-Short Form; CES-D, Center for Epidemiologic Studies Depression Scale; DASS-21, Depression Anxiety Stress Scales-21; HADS, Hospital Anxiety and Depression Scale; HAMD, Hamilton Depression Rating Scale; HRSD, Hamilton Rating Scale for Depression; PHQ, Primary Health Questionnaire; SCL-90, Symptom Checklist 90 (Revised); SMFQ, Short Mood and Feelings Questionnaire.*

##### Intervention and control conditions

Eight of the included studies adopted a standard 8-week MBSR program (Shapiro et al., 1998; Biegel et al., 2009; Kang et al., 2009; Raes et al., 2014; Aeamla-Or, 2015; Huang et al., 2015; Song and Lindquist, 2015; Johnson et al., 2016). Two studies adapted the program to a 12-week plan (Sibinga et al., 2013, 2016). One study adapted the program to an 11-week plan (Bluth et al., 2016) and one adapted the program to a 10-week plan (Hazlett-Stevens and Oren, 2017). Four studies used a 6-week MBSR program (Hindman, 2013; Dvořáková et al., 2017; Freedenberg et al., 2017; Shomaker et al., 2017) and two studies featured an MBSR program with 4 weeks (Jimenez, 2008; McIndoo, 2015). All 18 studies examined immediate effects at the end of intervention. Seven studies examined follow-up effect (i.e., 8, 12, 16, and 24 weeks) (Jimenez, 2008; Biegel et al., 2009; Raes et al., 2014; Aeamla-Or, 2015; McIndoo, 2015; Johnson et al., 2016; Shomaker et al., 2017).

Two studies compared MBSR plus TAU (i.e., standard medicine treatment and psychological therapy) with a TAU control group (Biegel et al., 2009; Huang et al., 2015). Six studies compared MBSR with active control conditions (Jimenez, 2008; Sibinga et al., 2013, 2016; Bluth et al., 2016; Freedenberg et al., 2017; Shomaker et al., 2017). Ten studies compared MBSR with no treatment (Shapiro et al., 1998; Kang et al., 2009; Hindman, 2013; Raes et al., 2014; Aeamla-Or, 2015; Song and Lindquist, 2015; Johnson et al., 2016; Dvořáková et al., 2017; Hazlett-Stevens and Oren, 2017).

##### Participant characteristics

Sample sizes of the included studies ranged from 23 to 408, with a total of 2,042 participants. Of these participants, the mean age was 17.2 (*SD* = 3.3). Three studies involved diagnoses of depression (Biegel et al., 2009; Huang et al., 2015; McIndoo, 2015). The remaining studies were conducted with youth displaying varying extents of depressive symptoms (Shapiro et al., 1998; Jimenez, 2008; Kang et al., 2009; Hindman, 2013; Sibinga et al., 2013, 2016; Raes et al., 2014; Aeamla-Or, 2015; Song and Lindquist, 2015; Bluth et al., 2016; Johnson et al., 2016; Dvořáková et al., 2017; Freedenberg et al., 2017; Hazlett-Stevens and Oren, 2017).

### Risk of Bias in Included Studies

**FIGURE 2:**
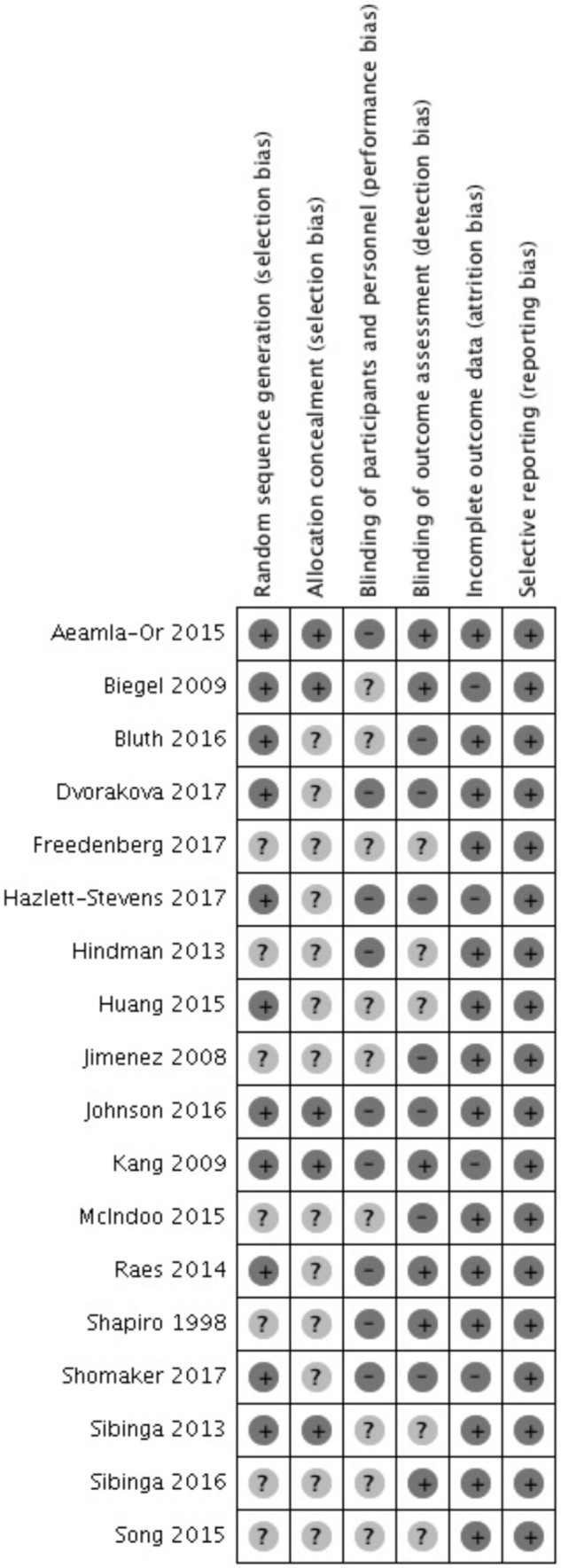
Risk of bias summary.

Sixty percent of the included studies had low risk of bias in sequence generation, and 28% of included studies had low risk of bias in allocation concealment. Randomization methods of the remaining studies were unclear, because they did not mention random sequence generation in their studies and did not explain how they allocated participants. Thirty-three percent of the included studies mentioned using blinded personnel or outcome assessors. Blinding methods for participants were not feasible, and it also turns out that all the included studies did not achieve this form of blinding. Of the included studies, 78% had low attrition bias because they had low dropout rate or mentioned using intention-to-treat analysis. No reporting bias was detected in included studies. A risk of bias summary is presented in **Figure 2**.

### Effects of Interventions

**FIGURE 3:**
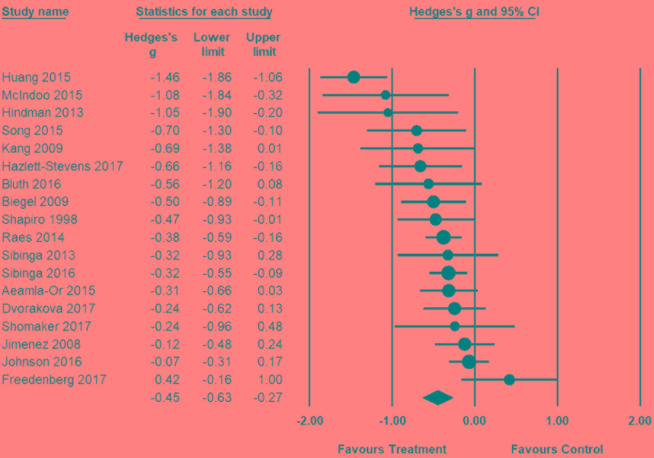
Posttest effect size of MBSR for depression.

Posttest effect size for these 18 studies comparing MBSR to controls (e.g., TAU, no intervention, and active control conditions) are shown in **Figure 3**. The combined posttest effect size was *g* = −0.45 (95% CI = −0.63, −0.27), indicating that MBSR had moderate effects in reducing depressive symptoms. The overall *I*^2^ indicated that 69% of the variability across studies was due to heterogeneity rather than chance. The *T* of 0.30 is the standard deviation of underlying effects across studies. The prediction interval is within −1.05 to 0.15, which suggests a large amount of inconsistency among effect sizes.

The combined follow-up effect size using robust variance estimation with 10 effect sizes from seven studies was *g* = −0.24 (95% CI = −0.54, 0.06), which indicates no statistically difference between the MBSR group and the control group in follow-up tests. The power to detect a population effect of 0.30 was 0.70. As such, the nonsignificant result must be interpreted in the context of the low statistical power.

#### Meta-Regression Analyses

Meta-regression results are presented in **Table 2**. We found that the average treatment effect was moderated by control condition (β = 0.37, SE = 0.18, *p* < 0.10), indicating that effects were larger for MBSR groups compared to no treatment or TAU conditions relative to active control groups. The average treatment effect size was also moderated by treatment duration, especially during the follow-up period (β = −0.41, SE = 0.15, *p* < 0.10). The effect size in studies among individuals diagnosed with clinical depression at baseline was larger than participants with various depressive symptoms only (β = −0.36, SE = 0.37, ns), but it did not reach a statistically significant level due to low statistical power. The statistically nonsignificant effects of moderators do not provide strong evidence of the absence of moderator effects, and results should be interpreted in the context of the low statistical power. We did not find a moderating effect of participants’ age group (β = −0.03, SE = 0.19, ns). Although the moderator analysis by age was also underpowered, the difference was trivial in magnitude.

**Table 2:** Results of meta-regression.

	k1	k2	Coefficient	SE
Baseline depression	29	18	-0.355	0.366
Age group^a^	29	18	-0.033	0.185
Control condition^b^	29	18	0.374*	0.184
Treatment duration^c^ (all)	29	18	-0.350*	0.183
Treatment duration^c^ (post-test effect sizes)	19	18	-0.236	0.211
Treatment duration^c^ (follow-up effect sizes)	10	7	-0.407*	0.151

*k1 = number of effect sizes, k2 = number of studies.*

*^a^Reference group was age < 18.*

*^b^Reference group was no treatment or treatment as usual.*

*^c^Reference group is treatment less than 8 weeks.*

*^∗^p < 0.10.*

### Sensitivity Analyses

Sensitivity analyses were conducted by excluding studies that may have had large effects on meta-analysis results (i.e., either being an outlier or having high or unclear risk of bias in multiple domains). After excluded seven studies that have high or unclear risk of bias in four out of the six domains, the combined Hedges’ *g* for the remaining 11 studies was −0.46 (95% CI = −0.72, −0.20) for posttest. Follow-up hedges’ *g* for the remaining four studies was −0.25 (95% CI = −0.75, 0.25). Both results were comparable with the original analyses. After excluded one more study with the extreme value, the combined Hedges’ *g* for the remaining 10 studies was −0.31 (95% CI = −0.46, −0.17, −0.14) for posttest and unchanged for follow-up.

### Assessment of Publication Bias

**FIGURE 4:**
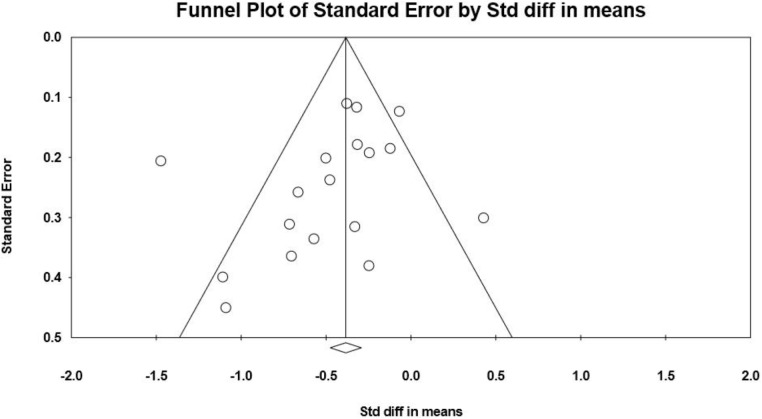
Funnel plot of standard errors by posttest effect sizes between MBSR and control.

**Figure 4** shows a funnel plot of posttest effect sizes in relation to the standard errors of the effect sizes. Eggers regression test showed no evidence of asymmetry in the funnel plot (intercept = −1.43; SE = 1.02; CI = −3.58, 0.73), and the trim-and-fill method indicated that no missing studies were needed to make the plot symmetric. However, due to the small number of included studies and the complicated nature of publication bias, we cannot conclude that our findings are robust against publication bias based on the funnel plot and the trim-and-fill method.

## Discussion

Eighteen RCTs involving 2,042 adolescents and young adults were assessed to examine the effect of MBSR on depressive symptoms. This review found a moderate posttest effect of MBSR in reducing depressive symptoms among adolescents and young adults when compared to control groups. This finding was generally consistent with previous reviews that found MBSR is moderately efficacious in treating mood disorders, including depression among children and adolescents in clinical and nonclinical settings (Zenner et al., 2014; Kallapiran et al., 2015). The current review did not find a significant follow-up effect of MBSR in comparison to control groups. Follow-up effect was also not synthesized in previous reviews. Due to a small number of included studies with follow-up tests and a lack of statistical power, we cannot draw a conclusion regarding the sustaining effect of MBSR in alleviating depressive symptoms for this population. Future clinical trials are encouraged to include multiple follow-up tests to examine the long-term effects of MBSR on youth depression.

Regarding meta-regression analyses, we found MBSR compared to no treatment or TAU may have greater effect sizes than compared to active control condition. This can be explained by the nature of the active control design, which helps to control alternative explanations for the possible effects of the intervention (e.g., attention from study staff, therapeutic environment, and social support; Kinser and Robins, 2013).

Meta-regression analyses also found that MBSR was efficacious for both clinical and nonclinical groups, which is consistent with prior systematic reviews that mindfulness-based interventions were efficacious in reducing depressive symptoms based on different samples of children and youth (Burke, 2010; Virgili, 2015; Zoogman et al., 2015). Previous review also found that MBSR was commonly used in nonclinical populations (Kallapiran et al., 2015). Although MBSR was originally developed in a clinical setting for patients (Kabat-Zinn, 1990), it is applicable to generally healthy individuals to reduce depressive symptoms and promote emotional well-being. Nevertheless, we found the overall effect size was larger among individuals with a depression diagnosis relative to the nonclinical group, which might be partially due to the low base rates of depression for the nonclinical group and floor effects (i.e., the relatively low baseline level of depression in the nonclinical group, leaving less opportunity for changes in depression scores). Findings were consistent with a recent study showing that the effect size of mindfulness interventions in clinical samples was larger than in nonclinical samples (Zoogman et al., 2015). Again, this finding should be interpreted with caution given the small number of studies included and the observational nature of moderator analysis.

In addition, the study found the longer the intervention duration, the larger the effect of MBSR on depressive symptoms, especially during the follow-up period. The finding was consistent with a previous meta-regression examining the effects of mindfulness-based therapies on a variety of psychological problems based on 182 studies, which found significant moderating effect of treatment duration (β = 0.01, SE = 0.00, *p* < 0.05; Khoury et al., 2013). However, the moderating effect of treatment duration on efficacy of MBSR in the literature is inconclusive. For example, Carmody et al. (2009) did not find a significant correlation between class contact hours and the mean effect size of MBSR interventions. The current review was the first to test the moderating effect of treatment duration on MBSR efficacy among young people. It is possible that young people may need more time to understand the meaning of MBSR and form practice habits in their daily life compared to adults. Especially over the long term, young people may lose interest in mindfulness practice and be less likely to persist if they did not get a solid practice foundation during the treatment period. Hence, it is understandable that MBSR training of 8 weeks or longer may have a greater influence on young people’s depressive symptoms during the follow-up period.

The present review had several limitations. First, our search only included published journal articles and dissertations in English and Chinese. Future research should include gray literature and research in other languages if possible. Second, relatively few RCTs on this topic were available and included in the current review, thus limiting the value of meta-regression. Due to the small number of studies with follow-up assessments, we were unable to draw a conclusion about the sustaining effect of MBSR for depressive symptoms among adolescents and young adults. We were also unable to perform a more sophisticated publication bias assessment. Third, many included studies have unclear risk of bias in allocation concealment and blinding. This is not uncommon for trials testing the efficacy for psychotherapies (Shean, 2014). To balance the potential risk of bias and the precision of the meta-analysis, we conducted sensitivity analysis by excluding studies with high or unclear risk of bias in multiple domains. The results of the meta-analysis should be interpreted cautiously considering the potential risk of bias. More rigorous RCTs with follow-up data (including short- and long-term) are needed to examine the effect of MBSR in treating depression among adolescents and young adults.

This study has potential implications for intervention. The moderate effect size of MBSR suggests that it is a promising approach in terms of reducing depressive symptoms and can be widely applied to treat depression or depressive symptoms among young people with various levels of depression severity, from expressing depressive symptoms to having a clinical diagnosis of depression. Given an increasing interest in positive education, MBSR that targets positive mental health could be incorporated into school-based educational programs to promote students’ emotional well-being. The study also found longer treatment duration (e.g., 8 weeks or more) is associated with larger follow-up effect size. This may suggest that the use of full-length MBSR may be necessary for adolescents and young adults to result in a larger sustaining effect.

## Author Contributions

XC, PZ, TL, AB, and IC contributed to problem formulation and study design. XC, AB, and TL conducted literature review, literature search and screening, data extraction, and data analysis. XC and AB interpreted the data and drafted the manuscript. XC, TL, AB, and IC critically revised the manuscript.

## Conflict of Interest Statement

The authors declare that the research was conducted in the absence of any commercial or financial relationships that could be construed as a potential conflict of interest.
